# Do Corticosteroids Still Have a Place in the Treatment of Chronic Pain?

**DOI:** 10.3389/fphar.2018.01229

**Published:** 2018-11-01

**Authors:** Nebojsa Nick Knezevic, Filip Jovanovic, Dimitry Voronov, Kenneth D. Candido

**Affiliations:** ^1^Department of Anesthesiology, Advocate Illinois Masonic Medical Center, Chicago, IL, United States; ^2^Department of Anesthesiology, University of Illinois, Chicago, IL, United States; ^3^Department of Surgery, University of Illinois, Chicago, IL, United States

**Keywords:** corticosteroids, chronic pain, osteoarthritis, back pain, neck pain

## Abstract

Corticosteroids have played a standard role in the multimodal pain management in the treatment of chronic spinal pain (cervical and lumbar) and osteoarthritis pain over the past three decades. In this review we discuss different types of injectable steroids that are mainly used for injection into the epidural space (for the treatment of radicular back and neck pain), and as intra-articular injections for different types of osteoarthritis related pain conditions. Furthermore, we discuss different approaches taken for epidural corticosteroid injections and spinal surgical rates when injections fail to resolve painful conditions, as well as the possibility of using local anesthetics alone for neuraxial injections, instead of in combination with corticosteroids. While we present some beneficial effects of newly available treatment options for low back pain and osteoarthritis pain, such as use of PRP and hyaluronic acid, corticosteroids remain important considerations in the management of these chronic pain conditions.

## Corticosteroids and Pain

While glucocorticoid steroids have historically been identified for centuries, the focus on their role in painful conditions has been incomplete. One rationale for this limited role could be our understanding that the benefits of the anti-inflammatory properties of steroids in pain management are exclusively supplementary to other therapies employed.

Glucocorticoids (cortisol/hydrocortisone) exert various physiologic effects primarily within immunological and metabolic systems, but also play a role in cardiovascular function and body fluid homeostasis. A healthy adult produces about 10–20 mg of cortisol daily, most of which is bound to corticosteroid-binding globulin, whereas exogenously administered dexamethasone is largely bound to albumin (Katzung, 2009).

The hypothalamic-pituitary-adrenal (HPA) axis plays a paradoxical role in regard to certain types of steroid responses to acute and chronic pain, which are dependent on dose, site, and mode of application of steroids. Pain comprehension is regulated at numerous levels of the central neuraxis, and particularly by higher cognitive processes. One study has shown that, upon injury, dorsal root ganglion (DRG) cells produce a neuropathic pain state from disinhibition of pain signal transmission, while glial cells prolong this condition through growth factor (GF) release and their subsequent action on the immune system (Fields, 2009). Future studies should focus on therapeutic alterations of glial-mediated hypersensitivity as well as on morphological and functional changes in important higher cerebral regions.

Oral, intramuscular, intravenous, transcutaneous, and neuraxial administration of corticosteroids has, over the past 30 years, been used in the management of different degenerative disease states (cervical and lumbar degenerative disease, osteoarthritis, etc.). During the pain management evolution from using oral steroids to fluoroscopically-guided epidural and transforaminal steroid injection techniques, research was begun to implement an algorithm for using the most superior methods of relieving back pain and radicular pain. In addition, corticosteroids are used intraarticularly for treating different osteoarthritis pain conditions. The aim of this review is to portray the evolution of the roles of steroids in pain management as well as to address the present debates among pain management specialists with respect to treatment options used in the management of chronic radicular type spinal pain, including the types of steroids and techniques performed. Moreover, special emphasis will be placed on the relationship of incorporating our literature review and formulating clinical decision-making, thereby acknowledging the need for identifying additional improvements in currently published pain management guidelines.

## Mechanism of Clinical Efficacy of Corticosteroids

The mechanism of action of corticosteroids is largely due to cytokine suppression. Risbud and Shapiro (2014) have assessed the relationship between cytokines and the development of intervertebral disc degeneration. Their proposed link between the two modalities begins with injury (i.e., trauma, infection, smoking) and follows with the release of cytokines from both the nucleus pulposus and annulus fibrosus as well from macrophages, neutrophils, and T cells. Cytokines include tumor necrosis factor alpha (TNFα), interleukin 1-beta (IL-1β), various other interleukins including IL-1 α/β, IL-2, IL-4, IL-6, IL-8, IL-10, IL-17, as well as IFN-γ, chemokines, and Prostaglandin E-2 (PGE-2). Proinflammatory cytokines enhance the activation and migration of immunocytes, with subsequent initiation of a molecular reaction, leading first to intervertebral disc degeneration and, ultimately, to a radicular back and/or neck pain.

Corticosteroids have direct and indirect roles in minimizing the production/release of previously mentioned cytokines by inhibiting Phospholipase A2 and the ensuing arachidonic acid metabolic pathway. The proposed mechanism results in both disc degeneration and pain expression reduction. Additionally, corticosteroids enhance the inhibition of transcription factors (e.g., NK-κB) and result in the subsequently decreased expression of pro-inflammatory genes, whereas upon binding to glucocorticoid responsive elements (GREs) adjacent to promoters of anti-inflammatory genes, they increase the expression of the latter.

## Different Injectable Steroids

A study by Haimovic and Beresford (1986) assessed the efficacy of oral dexamethasone in patients with lumbosacral radicular pain using a 7-day taper dose from 64 to 8 mg and showed negligible short- and long-term sciatica pain relief when compared to placebo. Webster et al. (2005) compared the initial approach of treating acute low back pain among 720 physicians of different medical specialists; it was found that among family medicine physicians, GPs, internal medicine physicians, as well as emergency medicine and osteopathic medicine specialists, nearly 25% of the surveyed physicians opted for systemic corticosteroids as their initial approach for the management of acute low back pain-related sciatica. Holve and Barkan (2008) evaluated whether oral prednisone could be used to treat acute sciatica. A 60–20 mg dose of prednisone was tapered for 9 days and was compared to placebo; upon weekly follow-ups in the first month, and monthly follow-up for 5 months, leg and back pain scores, use of analgesics, quality of life and functionality questionnaires demonstrated no significant benefit of early oral prednisone use in patients with sciatica pain (Holve and Barkan, 2008). With respect to their inadequate efficacy in decreasing low back pain, focus has been shifted from the use of oral corticosteroids to epidural steroid injections. Furthermore, the evidence has demonstrated that the use of steroid injections alone or in combination with other modalities has improved symptoms, treatment satisfaction scores and cost-effectiveness in the management of low back pain (Spijker-Huiges et al., 2014, 2015). Spijker-Huiges et al. (2014) demonstrated in a single-blinded, randomized controlled trial (RCT) improved treatment scores in a group of patients undergoing lumbar radicular syndrome treatment using segmental epidural steroid injections (SESIs) added to the usual pain treatments compared with control (*p* = 0.006). Another RCT demonstrated a significant improvement regarding the quality of life in the physical domain of the SF-36 questionnaire among patients utilizing SESIs compared to a control group in the management of lumbosacral radicular syndrome (Spijker-Huiges et al., 2015).

Depending on their water solubility and aggregation characteristics, various injectable steroid preparations can be broadly classified into two groups: particulate (“poorly soluble”) and non-particulate (“soluble”). In general terms, steroid names ending in “-lone” are particulate and long-acting, whereas those ending in “-sone” are non-particulate and short-acting. Particulate steroids (i.e., methylpredniso**lone** acetate, triamcino**lone** acetonide, and predniso**lone** acetate) have a longer duration of action and require fewer repeated injections than do soluble steroids; however, they may cause infarction of the brain and spinal cord if injected arterially. Non-particulates (soluble steroids) (i.e., betametha**sone** and dexametha**sone**) are arguably safer than particulates, but have short-lived anti-inflammatory effects. Tiso et al. (2004) presented a case report of a massive cerebellar infarction occurring in one of his own patients undergoing cervical transforaminal injection, and tested the hypothesis that particulate size in corticosteroid formulations may contribute to embolic vascular occlusion. They demonstrated that there were 8.6% of methylprednisolone acetate >50 μm and 3.7% of triamcinolone acetonide >50 μm in any given population as assessed using scanning electron microscopy. The implications of steroid size can be related to the diameter of arterioles and branching arteries wherein aggregates of particulate (insoluble) steroids could occlude these vascular pathways leading to a reduction or complete cessation of blood flow.

Kennedy et al. (2014) conducted a randomized, double-blind trial where they evaluated whether there was a significant difference regarding the effectiveness between particulate (triamcinolone) and non-particulate steroids (dexamethasone) when used in lumbar transforaminal epidural steroid injections in 78 patients (TFESI). Results demonstrated significant improvements with respect to pain and function at 2 weeks, 3 months, and 6 months with no apparent differences among different steroid preparations used (particulate/insoluble vs. non-particulate/soluble). However, a third TFESI was required to manage radicular pain more frequently in patients receiving dexamethasone than in patients receiving triamcinolone (17.1% vs. 2.7%, respectively) (7:1 factor) (Kennedy et al., 2014). Non-particulates/soluble steroids, on the contrary, have a decreased potential for infarction when compared to particulate steroids due to non-aggregation in end-arterioles, which may be one rationale for considering their use in transforaminal approaches.

## Preservatives in Corticosteroid Injections

In addition to their differences regarding chemical structure and particle size, steroids also vary with respect to different types of preservatives used in the manufacturing process to prolong their shelf lives. In a mutual collaboration, officials from the Centers for Disease Control and Prevention (CDC) and Food and Drug Administration (FDA) investigated a multistate outbreak of fungal meningitis and other infections in patients who had received contaminated, preservative-free methylprednisolone acetate (MPA) steroid injections made by one compounding company (Pettit et al., 2012). Even though it was assumed that a faulty manufacturing process might have been responsible (New England Compounding Center in Framingham, MA, United States), it also implicated the present challenges manufacturers face when making “preservative-free” glucocorticoid preparations. Consequentially, the majority of steroid preparations commercially prepared include preservatives (i.e., benzyl alcohol, polysorbate, monobasic sodium phosphate, polyethylene glycol, myristyl gamma picolinium chloride, benzalkonium chloride) for the purpose of sterility preservation and for enhanced shelf life. Despite the paucity of evidence regarding the clear risks with manufactured steroids with added preservatives shown in the fungal meningitis outbreak, the hazardous potential of added preservatives in commercially available MPA was noted in a study conducted by Knezevic et al. (2014a). This study demonstrated a linear dose-response relationship between increased concentrations of either PEG or myristyl-gamma-picolinium chloride or their combination and cytotoxic effects on dorsal root ganglia (DRG) sensory neurons in rat models. Candido et al. (2011) demonstrated a method of decreasing the concentration of polyethylene glycol (PEG) preservative in the commercial formulation of methylprednisolone acetate (MPA) injection. This study showed that by inverting the vial with a commercial formulation of MPA for about 2–4 h, prior to aspirating its contents, an average of 85% of PEG per vial would be removed (Candido et al., 2011).

## Which Approach Is Superior in Terms of Efficacy for Using Epidural Steroids in the Treatment of Unilateral Lumbar Radicular Pain? Transforaminal; Interlaminar; Caudal

Transforaminal epidural steroid injections (TFESIs), interlaminar epidural steroid injections (ILESIs) and caudal epidural injections remain the most extensively evaluated and utilized epidural injection techniques for managing lumbar radicular type pain. Three systematic reviews showed that for chronic unilateral radiculitis secondary to intervertebral disc disruption, the addition of corticosteroids to local anesthetics used alone for injection can increase the efficacy of all three approaches (Benyamin et al., 2012; Manchikanti et al., 2012; Parr et al., 2012). Parr et al. (2012) reviewed 16 studies with caudal epidural injection techniques and demonstrated good evidence regarding chronic pain alleviation secondary to disc herniation or radiculitis in the short- and long-term when a combination of local anesthetic and steroids was used. In addition, fair pain relief when local anesthetics were used alone was appreciated in spinal stenosis, axial discogenic pain and failed back surgery syndrome. Benyamin et al. (2012) conducted a systematic review that included 26 studies, which evaluated the effects of lumbar interlaminar epidural injections in the management of different types of chronic low back and extremity pain. The use of local anesthetics with steroids was associated with good and fair evidence in the management of radiculitis secondary to disc herniation and radiculitis secondary to spinal stenosis, respectively. Moreover, fair results were shown for axial pain without disc herniation when local anesthetics were used with or without steroids (Benyamin et al., 2012). A systematic review of 27 studies that assessed transforaminal epidural injections for the low back and lower extremity pain was conducted by Manchikanti et al. (2012). Using a combination of local anesthetics and steroids, there was a good evidence in the management of radiculitis secondary to disc herniation and fair evidence for radiculitis secondary to spinal stenosis. In contrast, the evidence was fair with the use of local anesthetics alone for transforaminal epidural injections to prevent surgery. However, there was limited evidence with the use of local anesthetics with or without steroids in the management of axial pain and post-surgery syndrome (Manchikanti et al., 2012).

There is conflicting data about which technique, ILESI or TFESI, is superior in the treatment of sciatica. Cohen et al. (2013), in a purely opinion piece non-systematic review and non-meta-analysis supported by 317 references opined in their review that transforaminal injections were more likely to produce positive results than interlaminar or caudal injections. Furthermore, subgroup analyses indicated that the likelihood of positive response for lumbar herniated disc was somewhat greater when compared to spinal stenosis or axial spinal pain. This inference was challenged in a systematic review and meta-analysis by Chang-Chien et al. (2014). Their analysis compared TFESI vs. ILESI under fluoroscopic guidance in the treatment of 506 patients with unilateral lumbosacral radicular pain. There was a non-clinically significant 15% difference in the favor of TFESI vs. ILESI at 2 weeks for pain relief, while no efficacy difference between the two techniques was documented at 1 or 6 months. Moreover, functional improvement was better in ILESI (56.4%) vs. TFESI groups (49.4%), although this result was also non-clinically significant (Chang-Chien et al., 2014).

The main characteristics of all RCTs that compared different approaches and different types of steroids are shown in Table 1.

**Table 1:** Randomized controlledtrials testing different approaches.

Author	Journal and year of publication	Type of study	Total number of patient	Study groups	Key finding(s)
Haimovic and Beresford	Neurology 1986	Prospective, double-blind	33	Dexamethasone (oral) vs. placebo	**Dexamethasone is not superior to placebo** for treating sciatica
Holve and Barkan	JABFM 2008	Double-blind, controlled	27	Prednisone (oral) vs. placebo	**Oral steroid medication in patients with sciatica had no significant effect** on most parameters studied
Candido et al.	Pain Physician 2013	Prospective, randomized, blinded study.	106	ILESI midline vs. PSILESI (epidural)	**PSILESI was more effective in targeting low back pain** with unilateral radicular pain secondary to degenerative lumbar disc disease. Pressure paresthesia occurring ipsilaterally correlates with pain relief and may therefore be used as a prognostic factor
Spijker-Huiges et al.	BMC Musculoskeletal Disorders 2014	Pragmatic, single-blinded, randomized controlled trial	63	Care as usual vs. epidural steroid injection	**Patients from the intervention group were significantly more satisfied with the received treatment**. Positive effect of SESIs on back pain, impairment and disability in acute LRS
Kennedy et al.	Pain Medicine 2014	Prospective, randomized, double-blind trial	78	Dexamethasone vs. triamcinolone (TFESI) (epidural)	**Dexamethasone appears to possess reasonably similar effectiveness when compared with triamcinolone**. However, the dexamethasone group received slightly more injections than the triamcinolone group to achieve the same outcomes
Spijker-Huiges et al.	Archives of Physical Medicine and Rehabilitation 2015	Pragmatic randomized controlled trial	50	Care as usual vs. epidural steroid injection	Both groups show improvement in physical domains (SF-36): **Intervention group scored better than control group**. Cost-effectiveness acceptability curve implies that utility of adding ESI to usual care is cost-effective at 80% without additional investment
Manchikanti et al.	International Journal of Medical Sciences 2015	Two randomized controlled trials	240	Local anesthetic vs. local anesthetic with a steroid (epidural)	The group with local anesthetic alone achieved significant pain relief and functional status improvement with a lumbar interlaminar and caudal approach in 72 and 54%, respectively. The group receiving a combination of local anesthetic and steroid had a significant response rate with lumbar interlaminar and caudal approach in 67 and 68%, respectively. This analysis demonstrated that **epidural injections with local anesthetic using lumbar interlaminar approach in the management of chronic low back pain, after excluding facet joint and SI joint pain, may be superior over a caudal approach**
Singla et al.	Pain Practice 2017	Prospective randomized open blinded end point (PROBE) study	40	Lidocaine and methylprednisolone vs. leukocyte-free PRP and calcium chloride (intra-articular sacroiliac joint injection)	Compared to patients taking steroids, pain intensity was significantly lower among patients taking PRP at 6 weeks and 3 months. In addition, the efficacy of steroid injection at 3 months was reduced in steroid group and PRP group by 25 and 90%, respectively. When other factors were controlled, **patients receiving PRP showed a reduction of VAS ≥ 50% from baseline**. Patients receiving steroids had SF-12 and MODQ scores improved for up to 4 weeks, but then declined at 3 months, whereas the scores in patients taking PRP improved up to 3 months

*ESI, epidural steroid injection; ILESI, interlaminar epidural steroid injection; LRS, lumbosacral radicular syndrome; MODQ, Modified Oswestry Disability Questionnaire; PRP, platelet-rich plasma; PSILESI, parasagittal interlaminar epidural steroid injection; SESIs, segmental epidural steroid injections; SF-36, 36-item short form survey; TFESI, transforaminal epidural steroid injection; VAS, visual analog scale.*

## Surgery Rates Affected by Different Injectable Techniques

It is necessary to determine whether epidural steroid injections used in an acute episode of radicular low back pain can prevent the need for spinal surgery. If the primary focus was on spinal surgery requirements, lumbar TFESI would initially be considered advantageous over other techniques. It is burdensome to compare studies to answer this question because of the different approaches (TFESI vs. ILESI), steroid preparations utilized (particulate vs. non-particulate) and patient communities (acute vs. chronic disease).

Riew et al. (2000) presented in their study that 29/55 patients with lumbar radicular pain did not require surgical intervention for their condition in a 13–28 months follow-up following selective nerve-root injection with bupivacaine and dexamethasone compared to bupivacaine only. There was a highly significant difference between the number of patients who opted to proceed with the surgery having used bupivacaine alone (18/27) vs. bupivacaine and betamethasone (8/28). Moreover, the patients who had received bupivacaine and betamethasone had significant alleviation of low-back pain as well as significant improvement in their scores on the questionnaires about treatment expectations.

Another study conducted to determine whether there were differences between TFESI using either dexamethasone or triamcinolone was a double-blinded prospective trial on 78 patients with a unilateral radicular pain from single level herniated nucleus pulposus (Kennedy et al., 2014). The surgical rates between dexamethasone and triamcinolone groups were comparable at 14.6 and 18.9%, respectively; and while both steroids resulted in significant improvements regarding pain and function at 2 weeks, 3 months, and 6 months, there were no apparent differences between the two groups. In addition, there was a significant difference between the number of injections received; the dexamethasone group received significantly more injections than the triamcinolone group to achieve the same results (17.1% vs. 2.7%, respectively) (Kennedy et al., 2014). Knezevic et al. (2014b) commented on the high surgery rate from the previous study and noticed how these differences may be a result of biased preference for proceeding to surgery when voicing opinions from different medical specialists (surgical vs. chronic pain specialists).

A prospective, randomized, blinded study conducted by Candido et al. (2013) compared midline and lateral parasagittal approaches of lumbar interlaminar epidural steroid injection (ILESI) in 106 patients with unilateral lumbosacral radiculopathic pain. Results have shown that even though ILESI with both approaches demonstrated statistically and clinically significant pain alleviation, using the lateral parasagittal approach showed clinically and statistically significantly longer pain relief, better quality of life scores, improvement in everyday functionality, and less pain medication utilization when compared to the midline approach. However, patients using the lateral parasagittal approach had significantly higher rates of ipsilateral pressure paresthesia during the injection phase of the steroid procedure, which correlated with pain relief and could therefore be used as a prognostic factor. This study also showed that the surgery rate at the one-year follow-up was only 4%, (Candido et al., 2013) in contrast to much higher percentage in the Kennedy et al. (2014) study that utilized the TFESI approach. While it may be true that there are difficulties extrapolating from results in possibly disparate patient subject groups between the respective studies, these studies emphasize the difficulties in making a consensus statement with respect to surgery rates.

## FDA Warning From April 23, 2014

On April 23, 2014 the FDA issued a safety announcement expressing concerns that epidural corticosteroid injections may be accompanied by rare, but serious adverse events, including vision impair, stroke, paralysis, and ultimately death (FDA, 2014). However, this announcement was criticized by the members of pain management community for two reasons. First, the supplemented references in this letter were strongly oriented toward the transforaminal approach (higher rates of vascular compromise) (14/17 FDA references were exclusively related to transforaminal epidural steroid injections), whereas no reference expressed concerns with the use of epidural steroid injections using a lumbar interlaminar approach. In addition, the majority of previously discussed adverse events were related with the injection of particulate steroids (Candido et al., 2014). Even though the FDA warning should be taken with the utmost importance, it should still be clarified that given the unequal properties of different epidural steroid injections, it is difficult to draw a conclusion that the generalized risks described by the FDA accompanies the use of interlaminar epidural steroid injections.

## Can We Use Only Local Anesthetics Instead of Combination of Local Anesthetics and Corticosteroids for Epidural Injections?

Zhai et al. (2017) conducted a meta-analysis of 10 RTCs (a total of 1111 patients) to evaluate the effects of local anesthetics alone or in combination with steroids in epidural injections in the management of various chronic low and lower extremity pain conditions. Results showed that the Numeric Rating Scale pain scores were significantly reduced in 40.2% of patients without steroids and 41.7% of patients with steroids by 4.12 and 4.09 scores (on a 11-point numeric pain rating scale), respectively. Furthermore, 40.7% of patients using local anesthetic alone and 39.8% of patients using a combination treatment reached significantly improved functional status. Oswestry Disability Indices (ODI) between the two groups were decreased by 12.37 and 14.5, respectively. The opioid intake in the two groups decreased from baseline by 16.92 MME (mg morphine equivalents) in patients that did not use the steroid combination and 8.81 mg in patients who did. Finally, the average number of procedures per year in the local anesthetic group was 3.68 ± 1.26, and 3.68 ± 1.17 in the combination group, while the average total pain relief per year was 32.64 ± 13.92 and 31.67 ± 13.17 weeks, respectively. This study has confirmed the similar results when using epidural injections with local anesthetic alone or with steroids in the management of patients with chronic low back and lower extremity pain.

Manchikanti et al. (2015) conducted a comparative analysis of efficacy of caudal and lumbar interlaminar approaches of epidural injections in the management of axial or discogenic low back pain. Two RCTs that involved 240 patients with chronic low back pain not caused by disc herniation, facet joint pain, or radiculitis and who received either local anesthetic alone or in combination with a steroid were followed up for 24 months. The group receiving local anesthetic alone achieved significant pain relief and functional status improvement with a lumbar interlaminar and caudal approach in 72 and 54%, respectively. The group receiving a combination of local anesthetic and steroid had a significant response rate with lumbar interlaminar and caudal approaches in 67 and 68%, respectively. This analysis demonstrated that epidural injections with local anesthetic using a lumbar interlaminar approach in the management of chronic low back pain, after excluding facet joint and SI joint pain, may be superior to a caudal approach.

## The Role of Platelet Rich Plasma (PRP) in Management of Back Pain

Singla et al. (2017) conducted a prospective randomized open blinded end point (PROBE) study testing the role of PRP in the treatment of low back pain. They allocated 40 patients diagnosed with sacroiliac joint (SIJ) pain into two groups; one group received 1.5 mL of methylprednisolone (40 mg/mL) and 1.5 mL of 2% lidocaine with 0.5 mL of saline, whereas another group received 3 mL of leukocyte-free platelet-rich plasma (PRP) with 0.5 mL of calcium chloride using an ultrasound-guided SIJ injection. Compared to patients taking steroids, pain intensity was significantly lower among patients receiving PRP at 6 weeks and 3 months. In addition, the efficacy of steroid injections at 3 months was reduced in the steroid group and PRP group by 25 and 90%, respectively. When other factors were controlled, patients receiving PRP showed a reduction of VAS ≥ 50% from baseline. Patients receiving steroids had SF-12 and MODQ scores improved for up to 4 weeks, but then declined at 3 months, whereas the scores in patients receiving PRP improved up to 3 months.

## Corticosteroids in Osteoarthritis

Tian et al. (2018) conducted a systematic review and meta-analysis of 4 RCTs regarding the efficacy and safety of intra-articular injection of methylprednisolone for pain reduction in 739 patients with knee osteoarthritis. Results revealed significant improvement with respect to WOMAC (Western Ontario and McMaster Universities Arthritis Index) pain scores and physical function at 4, 12, and 24 weeks when compared to placebo with no severe adverse events noted.

Juni et al. (2015) conducted a Cochrane meta-analysis of 27 RCTs to evaluate the benefits and harms of intra-articular corticosteroids in 1,767 patients with knee osteoarthritis. The use of steroids was both met with higher pain score reduction (1.0 cm difference on a 10-cm VAS scale) and also with more effective function improvement (a difference in functioning scores of −0.7 units on WOMAC disability scale) when compared to control. Not only did the quality of life among patients taking steroids remain the same, but they were also less likely to experience adverse events and withdraw because of them.

A phase 2, open-label study of 81 patients with knee osteoarthritis evaluated the pharmacokinetic properties of intra-articular (IA) triamcinolone acetonide (TA) delivered as an extended-release, microsphere-based formulation (FX006) versus a crystalline suspension (TAcs). In this study, Kraus et al. (2018) showed that TA synovial fluid concentrations following FX006 and TAcs were quantifiable through weeks 6 and 12, respectively. When compared to TAcs, microsphere-based TA delivery via a single IA injection resulted in prolonged SF joint concentration, diminished peak plasma levels, and reduced systemic TA exposure (Kraus et al., 2018). Conaghan et al. (2018) performed a phase-3, multicenter, double-blinded study of 484 patients with knee osteoarthritis comparing the benefits and safety profile of intra-articular injections of FX006, saline-solution placebo and TAcs. It was found that FX006 provided significant improvement (∼50%) in average-daily-pain (ADP)-intensity from baseline to week 12 compared with placebo, whereas improvements in osteoarthritis pain were not significant for FX006 compared with TAcs (Conaghan et al., 2018). The roles of corticosteroids as treatment modalities for knee osteoarthritis have been described in Table 2.

**Table 2:** The role of corticosteroids in the management of patients with knee osteoarthritis.

Author	Journal and year of publication	Type of study	Total number of patient	Study groups	Key finding(s)
Tian et al.	Medicine (Baltimore) 2018	Systematic review and meta-analysis	739	Methylprednisolone vs. placebo (intra-articular injection)	**Significant improvement using methylprednisolone compared to placebo** with respect to WOMAC (Western Ontario and McMaster Universities Arthritis Index) pain scores and physical function at 4, 12, and 24 weeks
Juni et al.	Cochrane Database of Systematic Reviews 2015	Meta-analysis	1,767	Any type of intra-articular corticosteroid vs. sham intra-articular corticosteroid and no intervention	**Steroids were superior to control** in terms of pain score reduction (1.0 cm VAS scale difference) as well as more effective function improvement (a difference of −0.7 units on WOMAC disability scale)
Kraus et al.	Osteoarthritis Cartilage 2018	Phase 2 open-label study	81	Single intra-articular injection of extended-release, microsphere-based formulation of triamcinolone acetonide (TA) (FX006) vs. crystalline suspension (TAcs)	**Microsphere-based TA injections resulted in prolonged synovial fluid joint concentration, diminished peak plasma levels, and reduced systemic TA exposure when compared to TAcs**
Conaghan et al.	The Journal of Bone and Joint Surgery 2018	Phase 3, multicenter, double-blinded study	484	Intra-articular injections of FX006 vs. TAcs vs. saline-solution placebo	**FX006 was better than placebo** with respect to significant improvement (∼50%) in average-daily-pain (ADP)-intensity from baseline to week 12
Hangody et al.	Cartilage 2018	Multicenter, double-blind clinical trial	368	Intra-articular injections of Monovisc (hyaluronic acid) vs. Cingal (hyaluronic acid plus triamcinolone hexacetonide) vs. saline	Clinical improvement from baseline was significantly greater compared to saline through 12 and 26 weeks. **At 1 and 3 weeks, Cingal was significantly better than Monovisc for most endpoints**, however, there was no difference between the two treatment modalities from 6 weeks through 26 weeks
Campbell et al.	Arthroscopy 2015	Meta-analyses	3,230	Intra-articular platelet-rich plasma (IA-PRP) vs. control (intra-articular hyaluronic acid or intra-articular placebo)	**PRP resulted in significant improvements in patient outcomes at 2 months through 12 months after injection.** Patients with less radiographic evidence benefited more with PRP treatment. IA-PRP injection led to significant improvements in patient outcomes (WOMAC score, IKDC score, Lequesne index) and led to greater increases in the pooled effect size versus treatment with control (HA or NS) at 6 months after injection
He et al.	International Journal of Surgery 2017	Meta-analysis	1,794	Intra-articular hyaluronic acid and intra-articular corticosteroids	**Patients taking steroids had better VAS pain scores up to 1 month after injection compared to patients taking hyaluronic acid; however, at 6 months the reverse was true.** With respect to WOMAC score at 6 months the hyaluronic acid group showed greater relative effect

*HA, hyaluronic acid; IKDC, International Knee Documentation Committee; NS, normal saline; VAS, visual analog scale.*

There are two ongoing clinical trials testing an extended-release triamcinolone (FX006). One trial is evaluating mean standardized change in synovial fluid volume at 6 weeks following single intra-articular injection of FX006 32 mg in patients with osteoarthritis of the knee (NCT03529942). The other study is measuring the concentration of triamcinolone acetonide in blood plasma through 12 weeks as well as the incidence of treatment emergent adverse events following the comparison of single intra-articular injections of FX006 32 mg vs. TAcs 40 mg in patients with osteoarthritis of the shoulder or hip (NCT03382262) (Clinicaltrial.gov, 2018). The roles of corticosteroids as treatment modalities for knee osteoarthritis have been described in Table 2.

## The Role of Hyaluronic Acid in Management of Osteoarthritis

Hangody et al. (2018) conducted a multicenter, double-blind clinical trial wherein they compared the efficacy and safety between intra-articular injections of Monovisc (hyaluronic acid), Cingal (hyaluronic acid plus triamcinolone hexacetonide), or saline in 368 patients with knee osteoarthritis. Clinical improvement from baseline was significantly greater compared to saline through 12 and 26 weeks. The use of Cingal demonstrated a WOMAC Pain reduction by 70% at 12 weeks and by 72% at 26 weeks. At 1 and 3 weeks, Cingal was significantly better than Monovisc for most endpoints; however, the two treatment modalities showed similar benefits from 6 weeks through 26 weeks (Hangody et al., 2018).

Campbell et al. (2015) conducted a study with three meta-analyses (a total of 3,230 patients) to compare intra-articular platelet-rich plasma (IA-PRP) versus control (intra-articular hyaluronic acid or intra-articular placebo) in the treatment of knee osteoarthritis. Utilization of PRP resulted in significant improvements in patient outcomes that commenced at 2 months and which were maintained for up to 12 months after injection. Furthermore, it was shown that patients with less radiographic evidence of arthritis benefited more from PRP treatment, whereas multiple PRP injections were associated with increased risk of self-limited local adverse reactions. All studies found that IA-PRP injection led to significant improvements in patient outcomes (WOMAC score, IKDC score, Lequesne index) and to greater increases in the pooled effect size versus treatment with control (HA or NS) at 6 months after injection.

He et al. (2017) performed a meta-analysis of 12 RCTs in order to compare efficacy and safety of intra-articular hyaluronic acid and intra-articular corticosteroids in 1,794 patients with knee osteoarthritis. Patients taking steroids had better VAS pain scores up to 1 month after injection compared to patients taking hyaluronic acid; however, at 6 months the reverse was true. At 3 months after the injection, VAS pain scores were equal between the two groups. With respect to WOMAC score, there were no significant differences at 3 months, whereas at 6 months the hyaluronic acid group showed greater relative effect. There was equal efficacy regarding the improvement of active range of knee flexion between the two groups at 3 and 6 months. Rescue medication use after treatment initiation and proportion of withdrawal for knee pain were similar between the two groups, however, topical adverse effects were more common in the hyaluronic acid group when compared to the corticosteroid group.

## Conclusion

Corticosteroids have become a standard part of the multimodal pain management algorithm in the treatment of back pain (cervical and lumbar) and osteoarthritis over the past three decades. There are many studies demonstrating the effectiveness of epidural corticosteroids in managing radicular low back and neck pain. However, some of the studies have shown that even the use of local anesthetics without corticosteroids may be beneficial for patients. Since the majority of patients require multiple injections over the course of their disease progression, it is imperative to be aware of all risks and benefits, patients’ safety, and cost-effectiveness of these respective procedures. Despite the presence of new treatment options, such as PRP, hyaluronic acid, etc., for back pain and osteoarthritis, steroids still have a prominent place in the management of these chronic pain conditions.

## Author Contributions

FJ and DV wrotethe manuscript. NK and KC revised the manuscript and made final corrections. All authors approved the final version of the manuscript.

## Conflict of Interest Statement

The authors declare that the research was conducted in the absence of any commercial or financial relationships that could be construed as a potential conflict of interest.
